# Strain Promotes Triple Negative Breast Cancer Proliferation and Migration Via VEGFR-2

**DOI:** 10.1007/s12195-025-00866-x

**Published:** 2025-09-10

**Authors:** Shalarria Cooper, Molly Matthews, Michael Knight, Sharmila Sridhar, Anna Sorace, Lalita A. Shevde, M. K. Sewell-Loftin

**Affiliations:** 1 Department of Biomedical Engineering, University of Alabama at Birmingham, Birmingham, AL 35294 USA; 2 Heersink School of Medicine, University of Alabama at Birmingham, Birmingham, AL 35233 USA; 3 Department of Radiology, University of Alabama at Birmingham, Birmingham, USA 35233; 4 O’Neal Comprehensive Cancer Center, University of Alabama at Birmingham, Birmingham, AL 35233 USA; 5 Department of Pathology, University of Alabama at Birmingham, Birmingham, USA 35233

**Keywords:** Tumor microenvironment, Triple negative breast cancer, Tumor mechanobiology, Microphysiological systems

## Abstract

**Introduction:**

Triple negative breast cancer (TNBC) has significantly worse outcomes compared to other subtypes. Strains in the tumor microenvironment (TME) generated by cancer-associated fibroblasts (CAFs) can regulate TNBC progression. Recent studies suggest that expression of VEGFR-2 on TNBC is linked to decreased survival, while our prior studies show strains activate VEGFR-2 to drive angiogenesis. We hypothesized that VEGFR-2 on TNBC can be mechanically activated to alter migration and proliferation.

**Methods:**

We utilized MDA-MB-231 TNBC cells loaded into the center chamber of a multi-microtissue TME model; opposing side chambers were loaded with CAFs or normal breast fibroblasts (NBFs). A second series of studies utilized magnetic beads to generate strains in the model without secretion of growth factors. Microtissues were analyzed for TNBC migration and proliferation via Ki67 staining.

**Results:**

TNBC cells migrated significantly more towards CAFs compared to NBFs (5×); TME models with magnetic beads showed a 2× increase in migration compared to no strain controls. TNBC cells treated with shRNA against VEGFR-2 demonstrated decreased overall migration but still significantly more towards CAFs vs. NBFs (2×). Proliferation analyses showed strain significantly increased Ki67 in control cells (10%+ vs. 28%+) but not in shVEGFR-2 TNBC (~ 10% all conditions).

**Discussion:**

These studies demonstrate that strain in the TME drives increased migration and proliferation of TNBC. Loss of VEGFR-2 suppresses migration and growth, even with mechanical stimulation. Therefore, our results suggest that mechanosignaling via VEGFR-2 on TNBC may regulate disease progression and potentially explain failure of anti-VEGFR-2 drugs in breast cancer patients.

**Supplementary Information:**

The online version contains supplementary material available at 10.1007/s12195-025-00866-x.

## Introduction

Triple negative breast cancer (TNBC) is the deadliest subtype of breast cancer, due to a lack of targeted therapies and higher risk of metastatic spread compared to other subtypes [1–3]. The TNBC subtype is defined by its lack of hormone receptors including estrogen receptor (ER), progesterone receptor (PR), and human epidermal growth factor receptor (HER2). Approximately 20% of all new breast cancer diagnoses each year are expected to be TNBC, and the 5-year mortality rate for this subtype of cancer is nearly 50% due to the increased metastatic spread [3]. Previous research has demonstrated that the TNBC subtype is highly migratory compared to ER+ and PR+ types with increased resistance to several classes of chemotherapies [4]. Moreover, studies demonstrate that the human TNBC cell line MDA-MB-231 can generate metastatic growths in brain, lungs, and livers of SCID mice [5, 6]. While the lack of targeted therapies is a critical issue for treating this disease, there is still little mechanistic understanding of why TNBC is so much more aggressive compared to other breast cancer subtypes. Many of the inherent cell behaviors related to proliferation and migration, associated with tumor growth and metastasis respectively, have mechanotransductive regulatory elements [7, 8]. Notably, recent research has suggested that there are mechanobiological regulators of TNBC behaviors leading to the observed increases in proliferation and migration behaviors as well as causing specific organotropism of metastatic spread [9]. Specifically, softer cells demonstrate gene expression patterns more like brain metastases, whereas stiffer cells gene expression mimics bone metastases. Models of compressive forces in the TME drive metastasis of breast cancer to bone microenvironments [10]. Improvements in our understanding of the mechanobiological environment of TNBC could help uncover how mechanical signaling patterns are regulated to drive cell behaviors in a more physiologically-relevant model to develop new strategies to target this disease. The tumor microenvironment (TME) is highly dysregulated compared to normal, healthy tissues. The TME is comprised of remodeled extracellular matrices (ECM) that include increased collagen, with larger fibrils that re-orient along the tumor periphery as the disease progresses [11–13]. Increases in TME stiffness are associated with worse outcomes and some studies suggest that even healthy, dense breast tissue may be associated with increased risk of TNBC and metastatic spread of breast cancer [14]. As the tumor progresses towards later, metastatic stages, the collagen fibrils are remodeled to align perpendicularly to the tumor mass, allowing for “highways” of ECM for cancer cells to migrate away from the primary tumor. In addition to the stiffer matrix, the TME also has increases in interstitial fluid pressure and shear stresses due to increases in leaky vasculature that grow in response to pro-angiogenic cues within the TME [13, 15, 16]. The shear flow acts as a mechanical stimulus to drive tumor cell migration, adhesion, and proliferation, as well as additional angiogenesis through mechanoreceptors including beta-1 integrins, calcium channels, death receptors, or receptor tyrosine kinases such as VEGFR-2 [17–20]. Increased compressive forces exist in the TME compared to adjacent healthy tissues due to the “capsule” effect generated as the tumor cells proliferate within the confined space of the primary site. The capsule can be formed through increases in ECM deposition and physical compression of cancer-associated fibroblasts (CAFs) [21, 22].

Studies by Barbarzan et al. demonstrated that CAF signaling through actomyosin contractility increase compression forces at the edge of tumors in vitro and in vivo. In addition to compressive forces, recent work has also suggested that CAFs generate tensile strains in the TME [23–26]. These tensile forces are caused by the increased contractility behaviors of CAFs compared to normal fibroblasts, most likely due to increases in alpha-smooth muscle actin expression and Snail1 [27–30]. Increased traction forces have also been observed for patient matched primary CAFs compared to normal fibroblasts on polyacrylamide gels [23]. Our work further suggested that these strains lead to increased angiogenesis and vasculogenesis in a 3D microtissue model through mechanical activation of vascular endothelial growth factor receptor 2 (VEGFR-2) at both tyrosine residues 1054/1059 and 1214 in endothelial cells [16]. These studies prompted us to optimize our 3D microtissue platform for mechanobiological studies to investigate CAF-generated strains on TNBC cell behaviors.

The VEGF/VEGFR-2 signaling axis is most associated with inducing and regulating angiogenesis or the growth of new blood vessels from pre-existing vasculature networks. Upon binding VEGF, VEGFR-2 can be phosphorylated at one of several tyrosine residues, including Y951, Y1054/1059, Y1175, and Y1214, which are each associated with specific downstream effectors [31–33]. Notably, differences in which residues are activated by VEGF binding or mechanical cues have been described by our lab and others [16, 34, 35]. For this study, Y1054/1059 and Y1214 phosphorylation events were investigated, representing residues primarily activated by ligand versus strain. Angiogenesis is necessary for tumor growth beyond a diameter of ~ 200 μm, beyond which diffusion of oxygen and nutrients is insufficient to sustain cells within the tumor core [36–39]; since angiogenesis is a hallmark of solid tumor progression, therapeutic strategies targeting this increased blood vessel growth have been pursued as far back as the 1980s [36]. The first anti-VEGF monoclonal antibody, bevacizumab, was approved for the treatment of breast, lung, and colorectal cancer in 2005. However, in breast cancer, the combination of bevacizumab and traditional chemotherapies led to little improvements in progression free survival for breast cancer patients, and the FDA revoked its approval as a metastatic breast cancer treatment in 2011 [40]. Interestingly, VEGFR-2 is aberrantly expressed on numerous epithelial cancers including breast and ovarian cancer cells [41–43]. Several of these studies suggest VEGFR-2 expression is associated with worse prognoses and more aggressive breast cancer cell behaviors. The expression of VEGFR-2 on TNBC may provide an alternative target for this highly deadly disease. However, the role of VEGFR-2 on regulation of specific TNBC behaviors is not well understood.

In this study, we leveraged a model of the TME to investigate how mechanical stimulation alters TNBC migration and proliferation through VEGFR-2 signaling. The multi-microtissue platform allows for specific control of biochemical signaling through control over diffusion and interstitial fluid flows, while permitting high spatiotemporal control over loading the microtissues. These studies demonstrate that mechanical cues, in the absence of stromal secreted growth factors, may play a role in TNBC progression. Specifically, TNBC with low VEGFR-2 expression showed decreased proliferation and partially inhibited migration when stimulated with strain. As anti-VEGF/VEGFR-2 drugs are of great clinical relevance for breast cancer, our work suggests mechanosignaling of VEGFR-2 may be why current drugs show limited efficacy in patients. Results from our studies can be used to develop additional strategies to target epithelial tumors that express VEGFR-2, with the ultimate goal of improving patient outcomes.

## Materials and Methods

### VEGFR-2 Expression Analysis

To characterize VEGFR-2 expression in breast cancer samples, we utilized a publicly available data repository and Kaplan-Meier curve interface [44]. We investigated expression levels of VEGFR-2 (Gene name: *kinase domain receptor, KDR*) in patients negative for ER, PR, and HER2 (n = 127). To examine how hormone receptor expression of VEGFR-2 related to patient survival, we reset the interface and included patients with ER+ and any PR/HER2 statuses (n > 1000). Finally, we compared these survival curves to ER+ only to mimic our cell line studies (n = 84). Overall survival was plotted in months. Tissue microarray slides were purchased from Tissuearray.com including TNBC samples and a non-TNBC controls with ER+, PR+, and/or HER2+ statuses. The slides were stained for VEGFR-2 (Cell Signaling Technologies, 2479), CD31 (Invitrogen, MA5-13188), or alpha-smooth muscle actin (Abcam, ab5649) using the Tissue Biorepository/Pathology Core Research Lab at the University of Alabama at Birmingham. Samples were imaged by the Digital Pathology Shared Facility at the Pathology Core and processed using a custom color classification code in MATLAB [45]. Individual histological sections were analyzed to determine percent positive staining area for each of the markers of interest. For each tissue core, six distinct regions of interest (ROIs) were manually selected for purple (nuclear) staining and brown (positive antibody) staining to determine percent positive staining area for each of the markers of interest.

### Cell Culture

To model human TNBC, we utilized MDA-MB-231 cells (ATCC, HTB-26). As a control, the ER+ MCF7 line (ATCC, HTB-22) was used in initial studies of VEGFR-2 signaling. Stromal cells included immortalized breast cancer-derived CAFs and normal breast fibroblasts (NBFs) previously isolated and further characterized by our lab [25, 46]. All cells were cultured in Dulbecco’s Modified Eagle’s Medium (Gibco; 11995065) supplemented with 10% Fetal Bovine Serum (FBS) (Gibco; 26140079), 1% l-Glutamine (Gibco; 25030149), 1% Sodium Pyruvate (Gibco; 11360070), 1% Penicillin-Streptomycin (Gibco; 15140-122), and 1% non-essential amino acids (Gibco; 11140-050). To fully investigate the role of VEGFR-2 in disease progression, we generated a knockdown (KD) line of both cell types via shRNA (Horizon Discovery, SMARTvector Lentiviral shRNA; V3SH7591-225708239; Target Sequence—TAGACTGGTAACTTTCATC). Puromycin (3 μg/mL) was added to the media to select the modified cells. An additional cell line using tdTomato (td-Tom) as a fluorescent reporter was generated to facilitate tracking in migration studies (Takara Bio, pLVX-tdTomato-C1 Vector, 632564). The receptor tyrosine kinase (RTK) inhibitor SU5416 (3 μM) was used to inhibit VEGFR-2 signaling, and bevacizumab (25 μg/mL) was used to inhibit soluble VEGF. All cells were incubated at 37 °C and 5% CO_2_. The cells were fed every 2 days until the flask reached ~ 80% confluency, at which point they were passaged using 0.25% trypsin.

### 3D Microtissue Ring Assay

Polydimethylsiloxane (PDMS) (Dow Corning, Sylgard 184) rings were punched from a 1 mm sheet with an inner diameter of 0.8 mm; this model was previously developed by our lab to generate a highly controlled 3D microtissue disk [25, 47]. Microtissues were formed by mixing fibrinogen, thrombin, and cell suspensions such that final disks contained 5 × 10^4^ cells encased in 10 mg/mL fibrin. The rings were fed with Endothelial Cell Growth Medium-2 (EGM-2) media (Lonza, CC-3162) every other day. In some studies, thrombin-coated magnetic beads were included in addition to cells. Briefly, 5 μm iron oxide magnetic beads with a tosyl-surface coating (Dynabeads, Thermofisher, 14013) were coated with 50 U/mL thrombin based on previously developed protocols [25, 48]. The thrombin-coating allows for effective, but not chemical, crosslinking of the beads into the gels during fibrin gelation. The result is that movement of the beads cause deformation of the matrix. The protocol has been developed and optimized to cause similar matrix deformation magnitudes (~ 5 μm) as CAFs [25]. These systems were stimulated via an external magnetic field on a rotator plate to generate mechanical strains on the order of CAF-generated matrix deformations, based on prior measurements that were found to be 4–6 μm [25]. On Day 7, rings were fixed using 10% neural buffered formalin for 20 min at room temperature, followed by washes with Phosphate Buffered Saline (PBS). Samples were permeabilized using PBS + 0.5% Tween-20 for 30 min at room temperature. Microtissues were blocked with 2% BSA in PBS with 0.1% Tween-20 (Abdil) before incubation with primary antibody, Ki67 (Abcam, ab16667; 1:1000) in Abdil at 4 °C overnight on a rotating platform. Samples were washed 4× with PBST (PBS + 0.1% Tween-20), then incubated within secondary antibody in Abdil overnight at 4 °C (Alexa Fluor 647 goat anti-mouse, ThermoFisher A21245, 1:500). Then rings were washed 4× in PBST, with the third wash containing 1:1000 DAPI. Samples were stored in PBST for imaging. Microtissues were imaged on an Olympus IX83 by acquiring 100 μm z-stacks, with 2 μm step size, then processed in FIJI. The process included subtracting the background from the image, conducting max z intensity projection, thresholding, and cell counting.

### 3D Microfluidic Device Assay

Our prior work has developed a 3D microfluidic device to model the TME and investigate the role of mechanics on angiogenesis [26]. The device consists of three tissue chambers connected via 20 μm ports that allow for communication between the chambers. Each tissue chamber has two fluidic lines, which control media distribution to each chamber and regulate diffusion of media between chambers. For cell-based migration studies, MDA--MB-231 cells were loaded into the center chamber at a concentration of 1.0 × 10^7^ cells/mL into a 10 mg/mL fibrin gel. In a similar manner, CAFs or NBFs were loaded into the left- or right-side chambers. For the device experiments exploring the effects of strain on migration without the presence of stromal cells, magnetic beads were loaded into one side chamber while the opposing side chamber had fibrin only (cell- and bead-free). All devices were fed with “outward flow” where media was supplied only to the center chamber and diffused equally into the side chambers. The outward flow regime was implemented specifically to isolate mechanical cues from biochemical signals, ensuring that only the mechanical stimulation provided by cells or pseudo-cells at the interfaces influenced the angiogenic response. This generated a microtissue model where equal interstitial shear forces occurred in both side chambers; moreover, the media feeding schema caused factors secreted by NBFs or CAFs to be diffused away from the interface with center chambers. Microtissue models were cultured for 14 days before fixing and imaging. Fixation and staining protocols were described previously [26]. After this process, devices are imaged as described above, stitched and analyzed using FIJI software [49].

### Strain Treatments

TNBC or ER+ cells were strained using a Flex-Cell system (FX-6000T) to investigate the changes in VEGFR-2 activation and expression. Cells were seeded at 4 × 10^5^ per well on a collagen-I coated uniaxial well plate (UF–4001C). After 24 h, these plates are transferred to the baseplate and introduced to oscillatory strains at 9% elongation at 0.3 Hz to mimic strains produced by CAF and to stimulate respiration rates [16, 50, 51]. Treatment groups included the following: no treatment (NT), additional exogenous Vascular Endothelial Growth Factor (VEGF) (+VEGF) of 25 ng/mL, strain (ε), and strain plus exogenous VEGF. After the cells received treatment, the samples were lysed for protein analysis using RIPA buffer with HALT protease and phosphatase inhibitor (1:100, ThermoScientific, 78441). All samples were run in triplicate.

### Western Blot

Samples were analyzed to determine activation of VEGFR-2 in MDA-MB-231 and MCF7 cells by measuring phosphorylation of VEGFR-2 at Y1214, a tyrosine residue associated with mechanoactivation of the receptor [16, 35]. For each sample, 50–75 μg of protein was loaded into wells in a 10% PAGE-SDS gel, using standard western blot protocols. Afterward, the samples were transferred to PVDF membranes (Fisher Scientific, IPVH85R) and then blocked in 5% BSA in TBST. Membranes were incubated in primary antibodies: for VEGFR-2 (1:1000; Cell Signaling Technology, 2479), pY1054/Y1059 VEGFR-2 (1:1000-1:200; Invitrogen, 44-1047G), pY1214 VEGFR-2 (1:1000-1:200; Invitrogen, 44-1052) overnight at 4 °C. β-actin (1:40,000; Sigma-Aldrich, A1978) was used as a loading control. The next day, the membranes were washed in TBST followed by being incubated in 5% milk in TBST with secondary antibody (Anti-rabbit IgG, HRP-linked, Anti-mouse IgG, HRP-linked Antibody 7074, 1:2000 or Anti-mouse IgG, HRP-linked Antibody, Cell Signaling Technologies 7076, 1:2000) for 2 h. Using ECL and Femto-ECL (100:1) (ThermoFisher 32,106 and 34,095), blots were imaged using a LiCor system (Odyssey XF) and later analyzed in FIJI using densitometry.

### Statistical Analysis

Data from all studies are reported as the average ± SEM with n = 3 replicates as a minimum. Studies with only two sample groups were compared via student’s t-test assuming unequal variances. Data sets for studies with more than two groups were first tested for normality using the Shapiro-Wilks test. If the data were normally distributed, a one-way ANOVA with post-hoc Tukey test was conducted to evaluate statistical significance. In the case that samples were not normally distributed, a Kruskal-Wallis test with post-hoc Dunn test was performed. Statistical analyses for experiments were run using the Real Statistics Excel plugin (Release 9.4) [Copyright (2013–2023) Charles Zaiontz. www.real-statistics.com]. Samples were statistically significant if the p-value was less than 0.05.

## Results

### VEGFR-2 Expression is Higher in TNBC Compared to Other Breast Cancer Subtypes and Associated with Worse Patient Outcomes

**Fig. 1:**
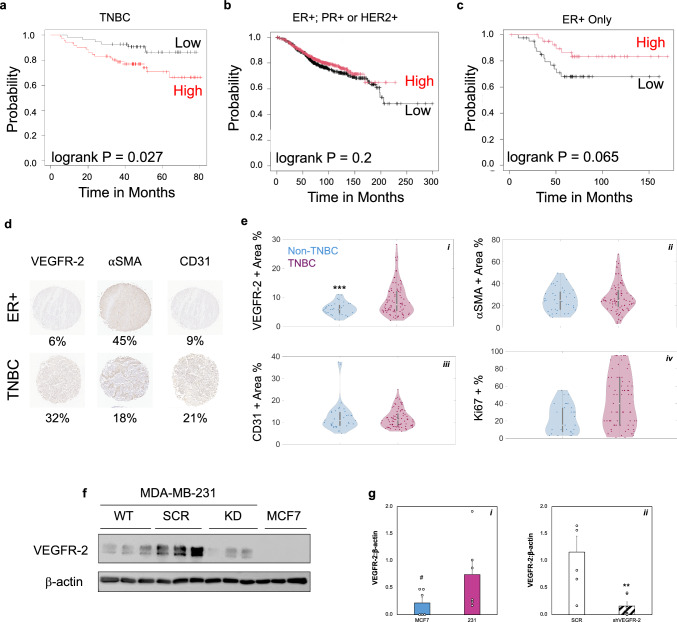
Increased VEGFR-2 in TNBC May Worsen Disease Progression.** a** Overall survival curve for 127 TNBC patients with Low (black) or High (red) expression of VEGFR-2, based on RNA-seq data from TCGA. Significant decreases in overall survival are observed for patients with high VEGFR-2 levels. **b** Overall survival curve for breast cancer patients with expression of ER+, PR+ and/or HER2+ showing VEGFR-2 gene expression. There are no significant differences for survival times in these patient populations (n = 1309 patients). **c** Overall survival for ER+ breast cancer patients (n = 84) showing no significant differences in survival times based on VEGFR-2 gene expression levels. Plots a-c generated with kmplotter.com 44 . **d** Representative images of IHC staining for VEGFR-2, αSMA, and CD31 in ER+ or TNBC breast cancer samples. Numbers underneath is area of positive staining for each shown slice, using MATLAB color recognition code 45. αSMA corresponds to stromal staining (CAFs or smooth muscle cells around vasculature); CD31 corresponds to vascular structures; VEGFR-2 staining is spread throughout tumor, stroma, and vascular structures in both tumor types. **e** (i–iii) Quantification of positively stained area for VEGFR-2 (i), αSMA (ii), or CD31 (iii) in patient samples. e-iv Ki67 positivity rate for samples shown in parts i-iii. N = (15–30); ***p < 0.001 vs. TNBC average. **f** Representative western blot images of MDA-MB-231 cell line and derived scramble (SCR) or VEGFR-2 knockdown (KD) lines. The ER+ MCF7 line is shown as a comparison. **g** Quantification of VEGFR-2 expression in cell lines, normalized to β-actin. (*i)* Comparison of MDA-MB-231, our model TNBC line to MCF7 cells, which are ER+ (^#^p < 0.05) (*ii*) Comparison of scramble (SCR) controls to the VEGFR-2 knockdown (shVEGFR-2 or KD) cells. The shVEGFR-2 KD line has ~ 80% decrease in VEGFR-2 expression compared to SCR controls (**p < 0.005), n = 6 replicates. Data shown as average + SEM, compared via Mann-Whitney tests. Full membrane images for all blots shown in Fig. S1

Higher gene expression levels of VEGFR-2 are associated with a significant decrease in overall survival for TNBC breast cancer patients (Fig. 1a); however, for breast cancer subtypes with ER+ and any PR/HER2 statuses, there appears to be no significant difference in survival times with respect to VEGFR-2 levels (Fig. 1b). Notably, high VEGFR-2 expression is not associated with statistical differences in outcomes for ER+ breast cancer patients (Fig. 1c). Western blot results indicate these cells express significantly lower protein levels of VEGFR-2 compared to the TNBC MDA-MB-231 cell line (Fig. 1g). While the patient population data reflects total gene expression levels, and is not restricted to tumor cells only, we believe this still supports that increased VEGFR-2 levels in TNBC may be related to disease progression and outcomes (Fig. 1c). To further explore and quantify expression of VEGFR-2 in TNBC, we examined a tissue microarray containing patient samples with both TNBC and receptor positive tissue sections. Using a MATLAB Color Classification module, our results demonstrate significantly higher VEGFR-2 staining present in TNBC tissues compared to all non-TNBC subtypes (Fig. 1e-i). There are no significant differences in CD31 (vascular) or αSMA (stromal) staining between these subtypes (Fig. 1e-ii,iii). While highly variable, the results show that Ki67 staining, indicating increased proliferation, is higher in TNBC samples versus non-TNBC ones (Fig. 1e-iv). As an in vitro model of TNBC, we quantified expression of VEGFR-2 via western blot in the MDA-MB-231 cells, and our results demonstrate ~ 4× higher levels in these cells compared to the ER+ MCF7 cells (Fig. 1f, g). To investigate the role of VEGFR-2 in our studies, we generated a VEGFR-2 knockdown (KD) line with shRNA that demonstrates ~ 80% decrease in VEGFR-2 protein compared to scramble (SCR controls) (Figs. 1f, g, S1). Altogether, this data demonstrates that TNBC presents upregulated VEGFR-2 compared to other subtypes and suggests that this may play a role in clinical progression and outcomes.

### Mechanical Stimulation Drives VEGFR-2 Activation in TNBC

**Fig. 2:**
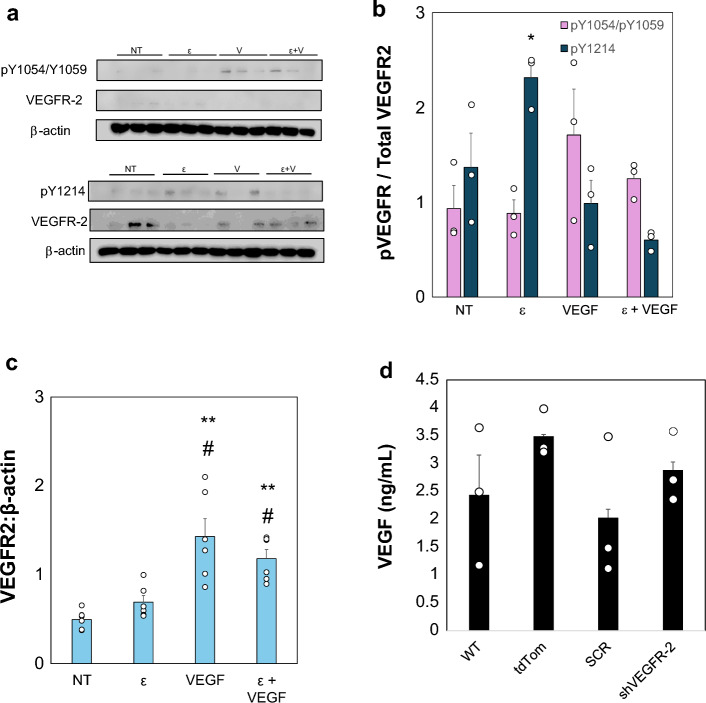
Strain Regulates VEGFR-2 Activation and Expression Levels in MDA-MB-231 cells.** a** Western blot images for phosphorylated MDA-MB-231 cells at Y1054/Y1059 or Y1214 residues. Total VEGFR-2 shown for same membrane. All samples were run with β-actin as a loading control in triplicate. Treatment groups included a control, No Treatment (NT), strain only (ε), exogeneous VEGF only (25 ng/mL, V) or strain plus exogeneous VEGF (ε + V). **b** Quantification of phosphorylation shown in a, normalized to total VEGFR-2 expression. Data suggests TNBC that express VEGFR-2 are sensitive to strain as observed via phosphorylation at Y1214 but not Y1054/1059. **c** Total VEGFR-2 expression for samples shown in a normalized to β-actin loading controls **d** Endogenous VEGF secretion was measured in MDA-MB-231 parental (Wild Type, WT), td-Tom (reporter line), or modified cells. Data was tested with ANOVA followed by post-hoc Tukey tests. All data shown as average + SEM for n = 3–6 replicates. ^#^p < 0.05 vs. Strain Only, **p < 0.01 vs NT. Full membrane images shown in Fig. S2

**Fig 3:**
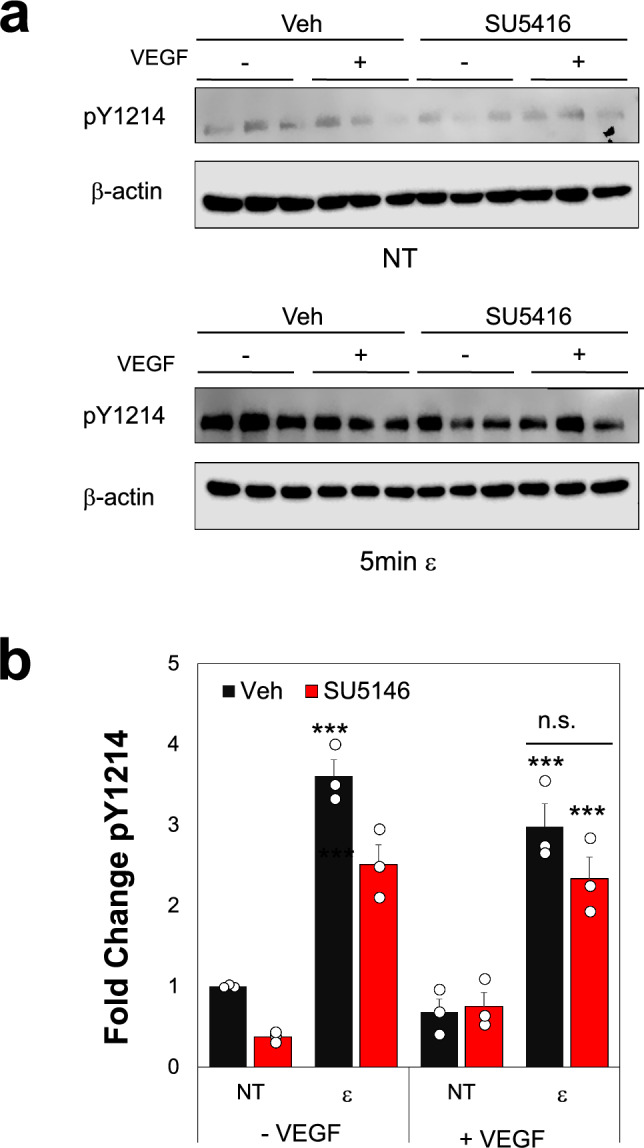
VEGFR-2 Inhibition Does Not Block Receptor Activation. **a** Western blot data for pY1214 with no treatment (NT) or 5 min strain (ε), with (+) or without (−) exogenous VEGF (25 ng/mL). The inhibitor or vehicle control was added at 3 μM during strain treatment. **b** Quantification of phosphorylation levels shown in part a, normalized to NT Veh controls. Data shown as average + SEM, n = 3 replicates. Samples compared with ANOVA followed by post-hoc Tukey test. ***p < 0.001 vs. non-strained (NT) with same VEGF treatment

Previous studies have suggested that VEGFR-2 can be activated via mechanical strains [16]. To assess if this phenomenon occurs in TNBC, we exposed MDA-MB-231 cells to uniaxial strain via a Flex-Cell system with and without exogenous VEGF. Our studies show cells had increased phosphorylation at Y1054/Y1059 with exogenous VEGF but not with any strain treatment (Figs. 2a, b, S2). The Y1214 residue shows significantly higher phosphorylation after 5 min strain treatment with no exogenous VEGF compared to no treatment (NT) controls. This suggests that mechanical strain promotes activation of VEGFR-2 via pY1214 in a manner that may be independent from ligand stimulation. Further, our data show that total VEGFR-2 levels were also significantly increased with exogenous VEGF, with or without strain (Fig. 2c). The ER+ MCF7 cells showed some increases in pY1214 expression with strain, but low VEGFR-2 expression for all strain or VEGF treatments (Fig. S3). To check for endogenous VEGF secretion by the TNBC cells and determine if this could impact our activation studies, we performed an ELISA. Results show similar amounts of secreted VEGF from all TNBC lines with little from endothelial cell controls (Fig. 2d). The levels of VEGF secretion from all MDA-MB-231 lines are about 10× lower than our exogenous treatments, suggesting that the endogenous levels were not driving differences seen in other studies. To further explore VEGFR-2 activation, we repeated the strain studies using a receptor tyrosine kinase inhibitor of VEGFR-2, SU5416. The data indicate that strain treatment increases pY1214 levels even in the presence of SU5416 (Fig. 3a, b). Moreover, there are no significant differences shown for pY1214 levels for SU5416 treated groups compared to Veh controls for strain treated TNBC, with or without exogenous VEGF. These findings imply that the activation of VEGFR-2 via mechanical strain is not substantially inhibited by SU5416, suggesting that the receptor mechanoactivation involves mechanisms that are distinct from those targeted by traditional RTK inhibitors. These results indicate that coupled ligand and mechanical stimulation drive enhanced VEGFR-2 activation in TNBC cells that RTK inhibitors do not block.

### VEGFR-2 Expression and Strain Drive Proliferation of TNBC

**Fig. 4:**
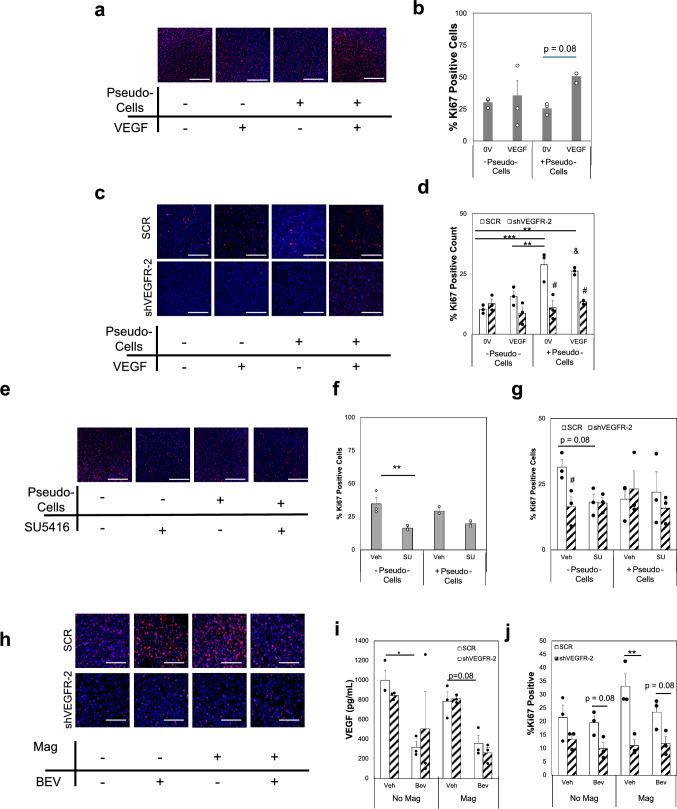
Mechanical Stimulation alters Proliferation via VEGFR-2 Signaling. **a** Representative IF images and quantification of td-Tom MDA-MB-231 cells embedded in 3D fibrin TME microtissues. **b** Quantification of proliferation via Ki67 expression in td-Tom 231 cells normalized to total cell count via DAPI stain. **c** Representative IF images of SCR or shVEGFR-2 knockdown (KD) MDA-MB-231 cells in microtissues. **d** Quantification of proliferation via Ki67 expression in SCR or shVEGFR-2 cells normalized to total cell count via DAPI stain. **e** Representative IF images and quantification of td-Tom, − 231 cells embedded in microtissues with inhibitor (SU5416) or vehicle control (Veh) treatments. **f** Quantification of proliferation in td-Tom cells treated with SU5416. **g** Quantification of proliferation in SCR or shVEGFR-2 231 cells normalized to total cell count. **h** Representative IF images of Ki67 staining of SCR or shVEGFR-2 231 cells treated with vehicle control (Veh) or 25 μg/mL bevacizumab (Bev). No Magnet samples were controls that included pseudo-cells but were not cultured above a rotating magnetic field, while Magnet indicates samples received strain from pseudo-cells. **i** VEGF ELISA results from conditioned media collected from microtissues shown in **h**. **j** Quantification of proliferation in SCR or shVEGFR-2 cells normalized to total cell count. In all images—Ki67 (red) and DAPI (blue). All samples in **a**–**g** were exposed to Strain via an external magnet placed above an orbital shaker. Exogenous VEGF treatment at 25 ng/mL. Scale bar = 500 μm. Data shown as average + SEM, n = 3 replicates. Samples were compared with ANOVA followed by post-hoc Tukey test (**b**, **d**) or Kruskal-Wallis followed by post-hoc Dunn’s test (**f**, **g**). ***p < 0.001, **p < 0.01; *p < 0.05; & p = 0.06 vs. SCR without pseudo cells but with exogenous VEGF. ^#^p < 0.05 vs SCR with same treatment. Note: Ki67 staining has been false colored to Red channel. Individual channel images are shown in Figs. S4 and S5

To investigate whether VEGFR-2 expression and mechanical stimulation promote changes in proliferation, TNBC cells were grown in 3D microtissue ring models. In some of the models, pseudo-cells (magnetic beads) were embedded with either MDA-MB-231 with td-Tom, SCR, or shVEGFR-2 (KD) cells. The magnetic beads, when stimulated with an external magnetic force, can cause matrix deformations or strains on the same order of magnitude as those driven by CAFs [25]. Results show that strain from pseudo-cells promoted proliferation (Fig. 4, shown in red, S4) in cells expressing VEGFR-2, even without exogenous VEGF (Fig. 4a–d). In samples where VEGFR-2 was knocked down, no significant changes in proliferation were seen regardless of the treatment group (Fig. 4c, d). In a separate series of microtissue studies, TNBC cells were treated with SU5416 before stimulation with pseudo-cells and/or VEGF. These studies showed that SU5416 can blunt proliferation responses compared to vehicle (Veh) controls in td-Tom or SCR MDA-MB-231 cells, regardless of mechanical stimulation (Fig. 4e–g). In these studies, the shVEGFR-2 samples displayed equivalent proliferation levels across all treatments, supporting that the inhibitor was not highly effecting cells with lower expression of VEGFR-2 (Figs. 4g, S5). Blocking VEGF through bevacizumab shows limited effects on proliferation, although decreased VEGF is observed in conditioned media from microtissues (Fig. 4h–j). Collectively, this data supports that VEGFR-2 on TNBC cells is responsive to matrix deformations or strains, driving proliferation even in the absence of exogenous VEGF. Moreover, interfering with VEGFR-2 signaling diminishes this response.

### TNBC Cells Migrate Towards Strain in a 3D Microfluid Device

**Fig. 5:**
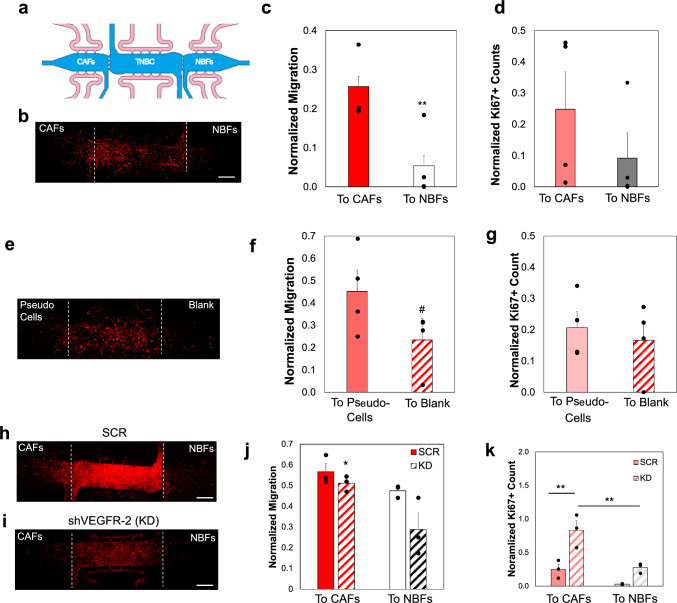
TNBC Cells Migrate Towards a Higher Strains in a Microfluidic Device. **a** Schematic of the microtissue platform for migration studies; insets specific which cell type was initially loaded into each region. Reprinted with permission via a Creative Commons License [26]. **b** Representative image of migration of td-Tom MDA-MB-231 cells after 14 days. **c** Quantification of number of td-Tom cells in side chambers normalized to total td-Tom signal in a center chamber for each device. **d** Proliferation quantification in side chambers, with Ki67 positive staining normalized to td-Tom signal in center chamber for specific devices. For data on fibroblast proliferation, see Fig. S3. **e** IF image of migration of td-Tom MDA-MB-231 cells into side chambers with pseudo-cells only or blank (bead free) chambers. **f** Quantification of total migration into side chambers for experiment shown in e, with cell count in side chambers normalized to total td-Tom signal in center chamber of specific device. ^#^p = 0.06. **g** Quantification of Ki67 positive cells in side chambers of devices from experiments shown in e. IF images can be seen in Fig. S4. **h**, **i** Representative images of SCR or shVEGFR-2 samples after 14 days, showing migration into side chambers. **j** Quantification of migration into side chambers for SCR or shVEGFR-2 cells, normalized to total RFP in center chambers for specific devices. **k** Quantification of proliferation in side chambers of devices shown from experiments in **g**, **h**. For fibroblast proliferation, see Figs. S6 and S7. In all images—White dashed lines are interfaces between chambers. Scale bar = 500 μm. Data shown as average + SEM, n ≥ 3 p devices per condition. Samples were compared with student’s t-test (**c**, **d**, **f**) or ANOVA followed by post-hoc Tukey test (**i**, **j**). *p < 0.05; **p < 0.01

A previously developed microfluidic device that accurately represents the TME was used to study the effects of CAF-generated strains on TNBC migration and proliferation. Microtissue models were loaded with the tumor cells in the center chamber, with stromal fibroblasts loaded into opposite side chambers (Fig. 5a). Previously, this model has shown that CAFs exert greater mechanical strains at the chamber interfaces due to the enhanced contractile activity, compared to NBFs [26]. The specific experimental configuration in these studies minimizes the influence of TNBC secreted factors to focus on mechanical stimulation by CAFs. TNBC cells were allowed to migrate to the side chambers for 14 days, and migration was quantified by normalizing red fluorescent signal in the side chambers to total levels in the center chamber for each device. Results showed that TNBC cells migrate significantly more toward the side with increased strain generated by CAFs (Fig. 5b, c). In these samples, proliferation increased in chambers with the td-Tom TNBC + CAFs compared to opposing td-Tom + NBF chambers (Fig. 5d). Further, separating out fibroblast proliferation from TNBC proliferation indicated that the TNBC in the CAF-containing chambers is higher as marked by increased Ki67 staining compared to NBF containing chambers (Fig. S6). In an additional set of studies that eliminated stromal secreted factors, devices that were loaded with pseudo-cells (magnetic beads) in one side chamber or blank controls in the opposing chamber. This experiment was designed to determine if increased migration or proliferation was due to CAF-secreted factors. Results showed that 231-td-Tom cells preferentially migrate toward chambers with pseudo-cells (Fig. 5e, f). To test the role of VEGFR-2 on TNBC migration, we utilized the SCR and shVEGFR-2 231 cell lines described above in the same TME model. Results showed that the devices with shVEGFR-2 (KD samples) displayed a decrease in overall migration towards either side chamber compared to SCR control samples (Fig. 5h-j). However, with both SCR and KD lines, TNBC cells still preferentially migrated towards the CAF-containing chambers. This observation suggests that while VEGFR-2 expression modulates the overall migratory capacity of TNBC cells, the inherent mechanical cues provided by CAF-generated strains continue to direct migration preferentially toward regions of higher contractile activity. High levels of proliferation were observed for SCR and shVEGFR-2 TNBC cells in the CAF containing chambers but not in the control NBF chambers (Figs. 5k, S7). Together, these studies suggest that mechanical stimulation driven by stromal features in the TME may promote enhanced TNBC migration and that regions with larger matrix deformations or strains are associated with increased proliferation.

## Discussion

In this study, we sought to determine the specific role of VEGFR-2 as a mechanoreceptor on TNBC cells in regulating migratory or proliferation behaviors. Previous studies have shown that VEGFR-2 is aberrantly expressed on epithelial tumor cells, and it may play a role in worse outcomes of breast cancer. However, no work had yet described whether this receptor is mechanically-activated in tumor cells and if VEGFR-2 signaling could drive functional changes in cell migration or growth. As VEGFR-2 is still a target in many cancer types, including TNBC, our work represents insights into the mechanobiology of TNBC that could uncover novel strategies to treat this disease. Our investigations united a 3D TME microtissue model, mechanical stimulation via stromal cells and pseudo-cells, and careful control over secreted factors to demonstrate the synergy between ligands and mechanical forces in VEGFR-2 signaling to promote cancer cell proliferation and migration.

Because tumor progression in solid epithelial cancers is often accompanied by increased angiogenesis within the TME, many anti-VEGF/VEGFR-2 therapies continue to be used as first-line treatments. While numerous cancer patients see benefits from such treatment, there is a significant gap in the substantial preclinical studies’ results and modest clinical improvements [52–54]. This discrepancy has promoted a multitude of studies into blocking angiogenesis in the TME or normalizing tumor-associated vasculature to attempt to create an environment where enhanced drug delivery can occur [55, 56]. The differences between mouse models used for developing such drugs and the human micro-mechanical environment are also quite large, and could potentially explain why promising therapeutics from early studies often fail to translate to clinical success [4, 52]. However, recent advances in modeling the TME using human microtissues such as the ones described here, permit investigations of human tumor and stroma with the added benefit of permitting micron-scale mechanical perturbations. MDA-MB-231 cells were transduced with shRNA lentivirus to generate a VEGFR-2 knockdown as well as a SCR control cell line. Notably, SCR cells displayed higher VEGFR-2 expression than WT. However, lentiviral transduction combined with puromycin selection has been shown to cause such enrichments as seen in Fig. 1f due to enhanced stress states [57, 58]. Our analysis therefore focuses on comparing the shVEGFR-2 to SCR controls, rather than unmodified cells. Our studies highlighted that VEGFR-2 highly expressed in models of TNBC compared to ER+ cells (Figs. 1f, g; S1), but that this corresponds to significant changes seen in clinical data for TNBC patients compared to receptor-positive cohorts (Fig. 1a–e). When looking at survival times, TNBC patients generally experience shorter overall survival compared to other populations, and higher VEGFR-2 gene expression appears to be linked partially to these poorer outcomes. The gene expression data presented in this paper represents global expression and is not exclusive to tumor cells only. Therefore, the survival curve data does not exclusively say that VEGFR-2 on TNBC is what is driving changes to survival times. However, higher expression of VEGFR-2 is also correlated with increased angiogenesis which is associated with worse outcomes in numerous cancer types [41, 42, 59, 60]. This is supported by somewhat higher staining for CD31, an endothelial marker indicating presence of blood vessels, in TNBC compared to non-TNBC in our histology studies (Fig. 1d, e-iii). These studies also supported that increased proliferation, as shown by higher Ki67 staining, occurs in TNBC samples (Fig. 1e-iv). Together, we believe this suggests that VEGFR-2 expression in the TNBC TME may be an important feature associated with aggressive disease. Furthermore, it presents a unique opportunity to target a receptor on multiple cell types in the TME of a highly aggressive disease subtype.

Previous studies in our lab have shown that VEGFR-2 on endothelial cells can be activated by CAF-induced strains in the matrix to promote angiogenesis [16, 26]. This pro-vascular behavior seems to be related to activation via tyrosine phosphorylation at Y1054/Y1059 and Y1214 which is not fully abrogated by inhibitors such as SU5416. While Y1214 has been shown be associated with a variety of different signaling pathways, such as ERK and p38, Y1054/1059 is needed for full kinase activity [33, 61, 62]. To determine if the same type of mechanical activation occurred in TNBC, we exposed MDA-MB-231 cells to strains using a 2D Flex-Cell system with and without exogeneous VEGF treatment. Strain studies indicated that activation at Y1214 in 231 cells, but not Y1054/Y1059, is driven by strain only in the absence of exogenous VEGF (Fig. 2a, b). Although total VEGFR-2 signal was low in certain treatments, this could reflect biologically relevant mechanisms such as receptor internalization or degradation [63]. Upon VEGF binding, VEGFR-2 is phosphorylation can trigger such internalization of the receptor from the cell surface. Additionally, mechanical stimulation may result in VEGFR-2 internalization promoted via SRC family kinases [64]. VEGF secretion was similar across the TNBC cells tested in our microtissue models at ~ 2 ng/mL (Fig. 2d). This was substantially lower than the 25 ng/mL of exogenous VEGF treatment tested in our strain studies and meant that differences in endogenous VEGF were not driving changes observed in migration or proliferation between the cell types. We conducted an additional experiment using bevacizumab to further investigate the role of soluble VEGF in our system in regulating TNBC proliferation (Fig. 4h). The result of this study shows that bevacizumab treatment reduces levels of soluble VEGF present in the conditioned media from microtissues containing MDB-MB-231 SCR and shVEGFR-2 and that mechanical stimulation does not alter soluble VEGF levels (Fig. 4i). Notably, bevacizumab treatment did not decrease proliferation for either SCR or shVEGFR-2 231 cells in the microtissues compared to vehicle controls (Fig. 4j).

Additionally, the mechanically-induced phosphorylation at Y1214 is not blocked when the 231 cells are treated with SU5416 and strain, with or without exogenous VEGF (Fig. 3a, b). In control studies using MCF7 cells, while there was some increase in pY1214 levels with strain treatment, it was still substantially less VEGFR-2 overall in these cells compared to the TNBC cells tested (Figs. S2, S3). Finally, exogeneous VEGF treatment and VEGF + strain elicited higher levels of total VEGFR-2 expression in the MDA-MB-231 cells while strain alone decreases this expression (Fig. 2c). As VEGFR-2 activation is typically followed by internalization and degradation in endothelial cells, it may be that tumor cells lack this same mechanism which limits feed-forward signaling [65]. Therefore, exposure to high levels of VEGF in the TME may prolong VEGFR-2 signaling in tumor cells in a manner that also limits effectiveness of VEGF/VEGFR-2 inhibitors. These studies only further highlight the complexity of mechanobiological crosstalk with ligand signaling in the TME that must be investigated for the development of next generation treatment strategies.

To determine how mechanosignaling via VEGFR-2 altered phenotypic behaviors of TNBC related to growth and metastasis, we investigated proliferation and migration in 3D TME models using our modified MDA-MB-231 cell lines. In studies using static microtissues called the ring assay, our results demonstrated that a combination of exogenous VEGF and mechanical stimulation via pseudo-cells drove higher levels of proliferation as observed by Ki67 staining in the td-Tom reporter line (Fig. 4a, b). In the SCR control line pseudo-cell mechanical stimulation alone, in the absence of exogenous VEGF, was sufficient to significantly increase proliferation. However, the shVEGFR-2 line showed lower levels of Ki67 staining in all treatment conditions, indicating a blunted proliferation response (Fig. 4c, d). In the VEGF-inhibition assay with bevacizumab (Bev), our results demonstrate decreases in soluble VEGF do not affect proliferation, regardless of VEGFR-2 levels or mechanical stimulation (Fig. 4h–j); the mechanism of action for bevacizumab is the binding of the VEGF ligand to prevent it from binding with its receptors [66]. Therefore, the effects of ligand binding should be greatly reduced in samples treated with bevacizumab. Even with this inhibition, our studies of mechanical stimulation show somewhat increased proliferation in SCR 231 cells with mechanical stimulation from pseudo-cells, suggesting that some mechanoactivation of the VEGFR-2 receptor may be occurring. While the role of ligand stimulation cannot be excluded fully, this data suggests that mechanical stimulation is partly able to contribute to VEGFR-2 mechanoactivation at least somewhat independently of ligand binding. Studies using SU5416 as an inhibitor of VEGFR-2 demonstrated that proliferation of TNBC can be blocked by soluble reagents. DMSO has been demonstrated to destabilize proteins and alter membrane fluidity, thus having the potential to disrupt many signaling pathways [67, 68]. This may potentially explain why there is somewhat of a decrease in proliferation in SCR samples that received Veh controls with mechanical stimulation from pseudo-cells (Fig. 4g). Comparing the shVEGFR-2 to SCR cells for this condition show no significant difference, indicating that the loss of VEGFR-2 may be protective against mechanically regulated side effects of DMSO. This can alter mechanoactivation which explains the inhibition of the pseudo cells in the SCR samples. These systems were stimulated using an external magnetic field on a rotator plate to generate mechanical strains of the same magnitude as CAF-generated matrix deformations. This is based on previously developed protocols where stimulation was tuned to match CAF-generated deformations on the order of ~ 5 μm [25]. These studies were designed to also mimic strain elongation settings from FlexCell studies.

Proliferation assays indicate that combinations of strain from pseudo-cells and exogenous VEGF moderately increase Ki67 expression in MDA-MB-231 cells after 7 days of stimulation (Fig. 4a, b), suggesting both ligand and mechanical stimulation drive this behavior. Proliferation studies on shVEGFR-2 cells indicate that these cells no longer respond to strain or exogenous VEGF alone or in combination but SCR controls demonstrate increased Ki67 when exposed to strain or VEGF (Fig. 4c, d). While phosphorylation data suggests strain or VEGF can increase differential activation of VEGFR-2 at different tyrosine residues, these studies were conducted in the acute setting at a much shorter time frame (5 min) than the proliferation studies (7 days). Therefore, our proliferation data reflects a longer-term regulation of cell behaviors. In addition, there are other tyrosine residues on VEGFR-2 known to be upstream of MAPK proliferation signaling [20, 69]. Together, our mechanistic studies and functional studies highlight that differential phosphorylation events can be regulated by strain or ligand stimulation and that this corresponds to functional changes in cell behaviors.

In studies with SU5416, MDA-MB-231 cells demonstrated decreased proliferation versus vehicle controls only when there was no mechanical stimulation (Fig. 4e). When stimulated with mechanical cues via pseudo-cells, there was no significant difference in Ki67 for nonmodified (WT) or SCR cells. As SU5416 is a broad receptor tyrosine kinase (RTK) inhibitor, it is possible that it is blocking proliferation promoted by other pathways and not exclusively VEGFR-2 signaling. However, the shVEGFR-2 cells showed no differences in Ki67 between vehicle and SU5416-treated samples (Fig. 4g). This suggests that blocking VEGFR-2 via SU5416 at least partially blocks proliferation supported by endogenous VEGF ligand-based signals but does not fully block proliferation promoted by mechanical stimulation of VEGFR-2. This could be explained by the differential phosphorylation sites that are activated by the different stimuli (Fig. 2). Together these studies indicate a potential mechanoreceptor behavior of VEGFR-2 on TNBC cells. Furthermore, the use of the magnetic beads as “stromal cells” to generate mechanical perturbations with the TME matrix highlights that strains can regulate proliferative behaviors alone, without stromal secreted growth factors. Such data may suggest why numerous promising therapeutic strategies investigated in preclinical models fail to yield significant clinical results, as the mouse biomechanical environment is dramatically different than what is found in human tissues.

Our microfluidic model mimicked a human TME that allowed for investigations of TNBC migratory changes with respect to VEGFR-2 signaling. Initial studies exhibited increased TNBC migration into chambers containing breast cancer-derived CAFs compared to regions with a patient-matched NBF (Fig. 5a–c and g–i). This increased migration towards CAFs was observed for all 231 lines tested, including the shVEGFR-2 knockdown line. These TME models were setup with “outward” flow, meaning that media was supplied at high pressures to the center chamber and then diffused into the side chambers at equal interstitial flow magnitudes. This flow pattern accomplishes two key controls within the model: (1) creating equivalent environments with respect to interstitial shear forces and (2) washing out secreted factors from the fibroblasts seeded in these chambers. Therefore, the primary difference between what is experienced by TNBC at the chamber interfaces is mechanical strain generated by the contractile behaviors of fibroblasts located at these interfaces. To further support this, we created the TME models using the pseudo-cells to generate mechanical stimulation in the form of strains at one chamber interface but not the opposing one; therefore, these systems had no differences in any endogenous secretion of growth factors from TNBC, CAFs, or NBFs. These studies demonstrated that the td-Tom 231 cells still preferentially migrate to the chamber where mechanical cues are higher, due to the stimulation with the pseudo-cells. Another advantage of this TME model is its capacity for parallel analyses. After completing the migration studies, devices were stained for Ki67 to assess whether the increased TNBC presence in the side chambers was driven primarily by cell migration or cell growth. To control for the presence of fibroblasts, FIJI was used to subtract co-localized staining to determine proliferation for each cell type individually. Studies show that ~ 30% of the cells proliferating in either of the side chambers are NBFs or CAFs (Fig. S6), so most of the proliferation observed is due to td-Tom TNBC cells. The only exception to this was samples with shVEGFR-2 TNBC in the NBF-containing side chambers; in these samples ~ 60% of the proliferating cells were NBFs and not shVEGFR-2 tumor cells. This could suggest that in the TME models with limited mechanical stimulation, there is overall less proliferation of TNBC even without VEGFR-2 signaling. Our studies showed that the TNBC that migrate into chambers containing CAFs seem more proliferative than TNBC in chambers with NBFs (Figs. 5g–i, S6). For studies with shVEGFR-2 cells, we observe that there appeared to be high levels of Ki67 staining in shVEGFR-2 TNBC cells in the CAF containing side chambers (Fig. 5j, S7d–f). The relatively large number of proliferating cells that should have limited expression of VEGFR-2 seems to oppose data in other studies described here. This could be attributed to a potential recovery of VEGFR-2 expression in these TNBC cells due to drift, as the TME models were cultured for 2 weeks without the puromycin selection agent. Overall, the data support that increased numbers of TNBC cells, through migration or proliferation, are present in the TME model with CAFs compared to microtissues loaded with NBFs. Also, while some of the TNBC proliferation could be due to the increased mechanical stimulation by CAFs through a variety of mechanoreceptors including integrins, CD44, and VEGFR-2, the current model does not prevent autocrine or juxtracrine signaling which could promote the enhanced proliferation we observed. While this is a limitation in the current model, the overall effect is the same: there is increased “tumor burden” represented in the microtissues with CAFs but not NBFs. Therefore, this model demonstrated that biomechanical stimulation of VEGFR-2 specifically expressed by TNBC cells can promote migration and may regulate proliferation in a 3D TME model.

## Conclusions

**Fig. 6:**
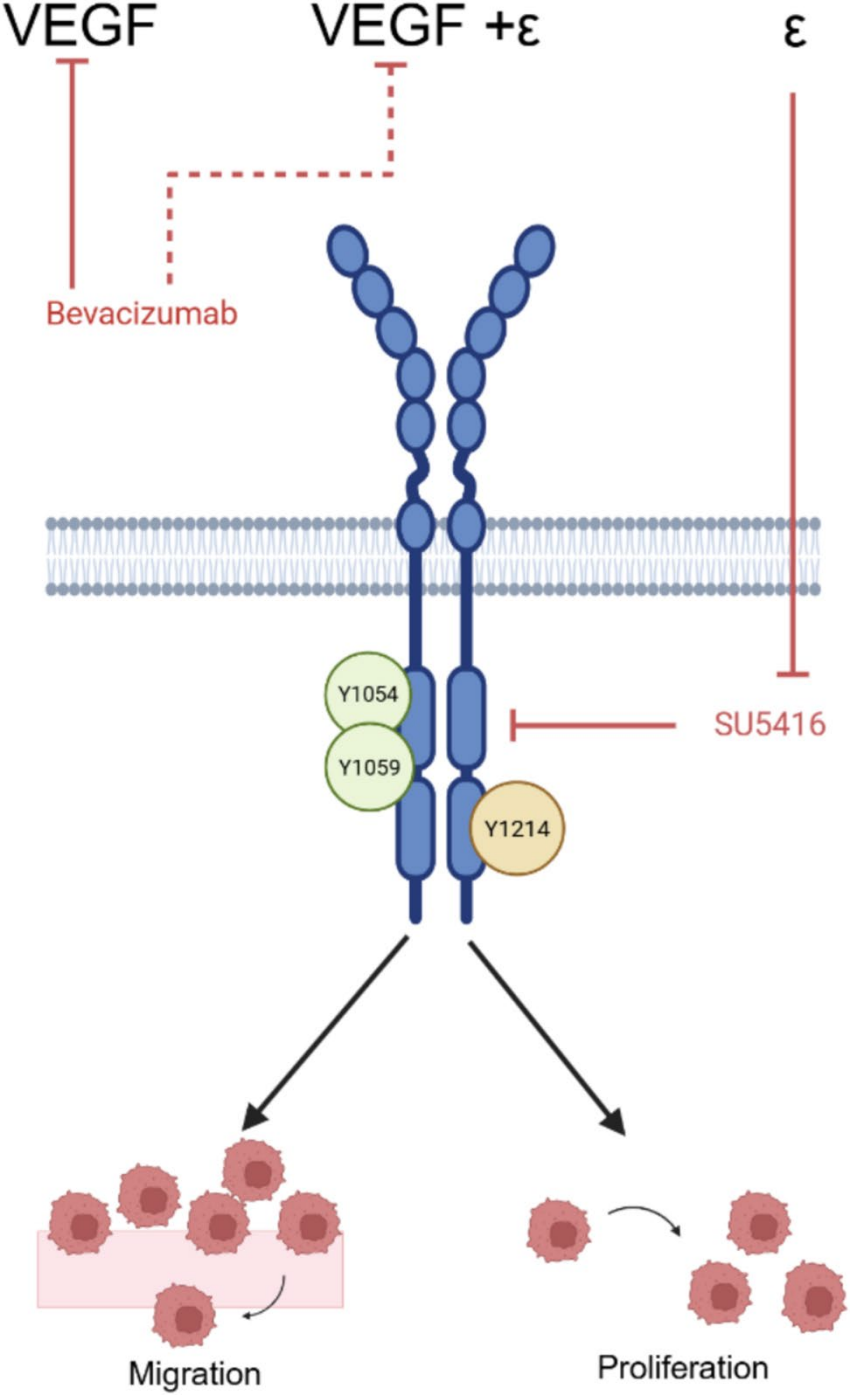
Strain and ligand dependent regulation of VEGFR-2 signaling drives TNBC cell responses. VEGF ligand binding to the VEGFR-2 receptor causes phosphorylation at Y1054/Y1059 and to a lesser extent Y1214. When VEGF and strain (ε) combine, activation is primarily observed at Y1054/Y1059. Bevacizumab specifically inhibits VEGF binding to VEGFR-2, but does not block the effects of strain. SU5416 as an RTK inhibitor blocks intercellular signaling related to VEGFR-2 phosphorylation. Strain activation of VEGFR-2 is associated with both increases in migration and proliferation, although combinatorial effects of VEGF + ε are also likely regulating changes in cell behaviors. Figure made with BioRender.

VEGFR-2 is upregulated in TNBC and, unfortunately, is associated with poor prognosis and decreased overall survival times. Anti-VEGF/VEGFR-2 treatments for breast cancer are not efficient at inducing improvements in disease progression. When tested in 3D microtissue models, TNBC cells containing VEGFR-2 exhibit high levels of migration and proliferation when exposed to strain from both CAFs and magnetic beads. Our mechanistic studies suggest that the Y1214 residue may be activated by strain alone, without exogenous VEGF stimulation. Furthermore, our studies suggest that different soluble inhibitors of VEGF/VEGFR-2 signaling may not fully block this pathway in the presence of strain stimulation or combinations of strain with exogenous VEGF (Fig. 6). Our data suggests that there is a correlation between increased migration and proliferation of cancer cells from mechanical stimulation. In conclusion, these studies highlight the importance of further investigating mechanical insights on cancer progression.

## Supplementary Information

Below is the link to the electronic supplementary material.

Supplementary file1 (DOCX 41301 KB)

## Data Availability

All datasets supporting the findings presented in this study are included in the article. Additional data supporting this study’s findings is available from the corresponding author upon reasonable request.
