# Supplementary material for: Strain Promotes Triple Negative Breast Cancer Proliferation and Migration Via VEGFR-2
Source: Cell Mol Bioeng. 2025 Sep 10;18(5):473–88. doi: 10.1007/s12195-025-00866-x (PMC12579648; doi:10.1007/s12195-025-00866-x)
Supplement: Supplementary file 1 — Supplementary file1 (DOCX 41301 KB) [file 12195_2025_866_MOESM1_ESM.docx]

**Fig. S1. Full Membrane Images for Western Blots showing validation of VEGFR-2 Knockdown in Triple Negative Breast Cancer Cells.** These are full membrane images that correspond to the data shown in Fig 1f,g (MCF7. WT, SCR, KD). Images on Left correspond to a single technical replicated (3 biological replicates per line). Images on Top Right are second technical replicate (3 biological replicates per line) shown with ladder and overexposure to show membrane edges). Bottom Left is images from Top without ladder and overexposure.

**Fig. S2. Full Membrane Images for Western Blots showing pY1054/1059, pY1214, and VEGFR-2 Expression Levels.** These are full membrane images that correspond to cropped data shown in Fig. 2a

**Fig. S3 VEGFR-2 Activation and Expression in ER+ MCF7 cells.** Western blots showing phosphorylation at Y1214 for MCF7 cells with Strain and/or exogenous VEGF treatment for 5min. For all samples, β-actin was used as a loading control.

**Fig. S4 Proliferation in Modified TNBC Cells with VEGFR-2 Inhibition.** Representative images of SCR or shVEGFR-2 231 cells showing Ki67 (red) and DAPI (blue). Corresponds to data show in Fig. 4g. Scale bar = 500μm.

**Fig. S5. Individual channel images for all proliferation studies shown in Fig. 4. For** all images, Ki67 has been false-colored red and DAPI is shown as blue. Scale bars = 500mm. (a,c) td-Tom MDA-MB-231 cells; (b,d) SCR or shVEGFR-2 MDA-MB-231 cells. (c) Corresponds to image shown in Fig. S4.

**Fig. S6 Breakdown of Fibroblast and TNBC Proliferation in TME Models.** **a** Representative IF images of Ki67 staining (yellow) shown for side chambers of device in 5b. Scale bar = 500μm. **b** Data from Fig. 5d shown with fibroblast proliferation rate (green, lower bar) and TNBC proliferation rate (red, upper bar) shown together. Total Ki67+ data normalized to RFP expression in Center chamber for a specific device. Data shown as average + SEM for n=4 devices. ** p<0.01 vs “To CAFs” data. **c** Individual replicate data shown for all devices for CAF proliferation (green) with TNBC proliferation (red) in side chambers loaded with CAFs. **d** Individual replicate data for NBF proliferation and TNBC proliferation in side chambers loaded with NBFs. Data in **c** and **d** shown as percentage of total number of proliferating cells in specific side chambers. **e,f** Representative IF images of Ki67 (yellow) staining in device shown in Fig. 5e. Scale bar = 500μm.

**Fig. S7 Breakdown of Fibroblast and Modified TNBC Proliferation in TME Models.** **a** Representative IF images of Ki67 (yellow) staining in device shown in Fig. 5h. **b** Data from Fig. 5j shown with fibroblast proliferation rate (green, lower bar) and SCR TNBC proliferation rate (red, upper bar) shown together. Total Ki67+ data normalized to RFP expression in Center chamber for a specific device. Data shown as average + SEM for n=3 devices. ** p<0.01 vs “To CAFs” data. **c** Individual replicate data shown for all devices for CAF proliferation (green) with SCR TNBC proliferation (red) in side chambers loaded with CAFs. **d** Individual replicate data for NBF proliferation and SCR TNBC proliferation in side chambers loaded with NBFs. **e** Representative IF images of Ki67 (yellow) staining in device shown in Fig. 5i. **f** Data from Fig. 5j shown with fibroblast proliferation rate (green, lower bar) and shVEGFR-2 (KD) TNBC proliferation rate (red, upper bar) shown together. **g** Individual replicate data shown for all devices for CAF proliferation (green) with shVEGFR-2 TNBC proliferation (red) in side chambers loaded with CAFs. **h** Individual replicate data for NBF proliferation and shVEGFR-2 TNBC proliferation in side chambers loaded with NBFs. Total Ki67+ data normalized to RFP expression in Center chamber for a specific device. Data shown as average + SEM for n=3 devices. ** p<0.01 vs “To CAFs” data. e and f Individual device data for shVEGFR-2 TNBC devices showing relative proliferation for fibroblasts compared to tumor cells. Data in b,c, e, and f shown as percentage of total number of proliferating cells in specific side chambers. All scale bars = 500μm.
